# Research note: Transient behavioral effects of feed restriction in a white feathered laying hen pullet strain - Assessment of frustration and locomotor energy expenditure

**DOI:** 10.1016/j.psj.2026.106690

**Published:** 2026-02-23

**Authors:** Ana K. Rentsch, Sachin Gautamadasa, Charlene Hanlon, Grégoy Y. Bédécarrats

**Affiliations:** a Department of Animal Bioscience, University of Guelph, Guelph, ON, Canada; b Campbell Centre for the Study of Animal Welfare, University of Guelph, Guelph, ON, Canada; c Department of Poultry Science, Auburn University, Auburn, AL, United States

**Keywords:** Abnormal pecking behavior, Aggressive behavior, Vertical locomotion, Animal welfare, Quantitative feed restriction

## Abstract

Quantitative feed restriction is used to effectively manipulate layer pullet growth trajectories in research and rarely in commercial practice. In this study, we asked whether this intervention impairs pullet welfare or affects locomotor energy expenditure. Pullets were raised in furnished floor pens in a split-plot design: Two ages at photostimulation (18 weeks: **early-PS** vs 20 weeks: **late-PS**) with two feeding regimens each (*ad libitum*: **AL** vs 20%-restricted: **R**) between 12 and 21 weeks. Home pen video recordings were annotated for four behavioral categories: abnormal pecking, aggression, high-energy aerial locomotion, and low-energy locomotion via ramps. Feed restriction increased abnormal pecking behavior at 16, 17, and 18 weeks (*P* = 0.0002). Aggression increased during puberty, which occurred sooner for AL and early-PS than R and late-PS pullets, then decreased (*P* = 0.016). In R pullets, abnormal pecking tended to be higher, and aggression was higher post-meal than pre-meal (*P* = 0.06 and *P* = 0.006). No effect of restricted feeding was detected on high- and low-energy vertical locomotion (*P* = 0.954 and *P* = 0.268). These findings indicate that quantitative feed restriction of layer pullets can be used for research with appropriate caution, though potential effects on latent feather-pecking risk and late-life bone health should be considered.

## Introduction

It is widely recognized that using severe feed restriction (up to 75% of *ad libitum* intake) to slow the growth of broiler breeder pullets (adolescent hens) results in **abnormal pecking** and increased **aggressive** behavior, indicating poor welfare (De Jong and Guémené, 2011). In layers (egg-type chickens), feed restriction can modify body weight, flock uniformity, and performance (Anene et al., 2023) and in research, it is used to experimentally manipulate growth trajectory to pursue developmental questions (e.g., Bahry et al., 2023 reduced body weight by 20%). However, research on feed-restricted layer pullet behavior and welfare is lacking and the differential selection in broilers (increased appetite) versus layers (egg production) makes direct comparisons inappropriate. Additionally, the structural complexity of layer and broiler breeder housing differs dramatically, introducing a new consideration for feed management.

In non-cage layer housing, chickens are challenged to locomote in a 3-dimensional housing structure. As ground-dwelling birds, chickens are highly motivated to seek out elevation for perching and night-time roosting (Olsson and Keeling, 2002). Layers use different modes of vertical locomotion to move between vertical structures: **aerial locomotion** – either wing-assisted (flying) or not (jumping), or **by ramp** – either walking or wing-assisted incline running (WAIR). From studies in other birds, it is known that flight is more energy demanding than walking (zebra finches: Bautista et al., 2001), even short, controlled flights (starlings: Nudds and Bryant, 2000).

In this study, we asked whether quantitative feed restriction impairs pullet welfare or affects locomotor energy expenditure. Feed restriction may induce frustration due to the inability to satisfy the motivational state of hunger. We expected this frustration to lead to an increase in abnormal pecking behavior and aggression. Feed restriction may also result in less energy, altering pullets’ expenditure. Hence, we expected feed-restricted pullets to favour low-energy over high-energy modes of vertical locomotion.

## Methods and materials

Ethical use of animals was reviewed by the Animal Care Committee of the University of Guelph (Animal Utilization Protocol # 5066).

Six hundred Lohmann LSL-Lite pullets, a widely used layer strain in North America, were moved from a pullet aviary (access to perches, platforms, and litter) to 24 experimental pens (44.4 m^2^) across four rooms at 12 weeks of age (WoA). Each pen was furnished with two elevated perches (320 cm perch space) and platforms (10,800 cm^2^ platform space), litter, nipple drinkers, and a feeder (300 cm of feeder space). Six nestboxes were added at 16 WoA. The initial group size of 25 pullets per pen was reduced stepwise when one bird per pen was euthanized for organ collection at 14, 16, 18, 19, 20, 21, and 22 WoA. Data from these dissections will not be presented in this paper. Rooms were kept at a consistent temperature of 20°Celsius.

Treatments were assigned according to a split-plot design, with two ages at photostimulation as the main plot factor (**PS**: 2 rooms/ age), and two feeding regimens as the subplot factor (3 pens/ room/ regimen). The feeding regimens were *ad libitum* (**AL**) or 20%-restricted (**R**) between 14 and 21 WoA, reducing body weight by 10% at 20 WoA. At PS (18 or 20 WoA), the light period was increased from 8 h (- 1500, 20 min of dusk and dawn) to 12 h by adding four hours at the end of the day and increasing light intensity from 4 to 6 to 10-15 Lux. Photoperiod was further increased over two weeks to reach 14 h light. R birds were fed twice daily at 0800 and 1300 pre-PS, at 0800 and 1500 post-PS. Pens were video recorded for two consecutive days weekly between 15 and 25 WoA. Videos of weeks 15 - 22 were annotated for behavioral indicators of frustration and locomotory energy expenditure for five continuous minutes, one hour before and after mealtimes (20 minutes/ day, 40 minutes/ week). A single observer developed the ethogram through repeated intra-observer reliability assessments until kappa was > 0.9 for the following behavior categories:

• **Abnormal pecking**: Object pecking (walls, perch, empty feeder), severe feather pecking, tissue pecking.
• **Aggressive behavior**: Aggressive pecking, sparring, aggressive displacement from the feeder.
• **Aerial locomotion**: jumping, flying.
• **Ramp use**: walking or running on the ramp.

Following preliminary analyses, it was decided to annotate additional time points (23 to 25 weeks) for aggressive behavior, to assess whether the apparent changes around maturation persisted, reversed, or stabilized with age.

All videos were annotated using the Behavioral Observation Research Interactive Software (BORIS, DOI: 10.1111/2041-210X.12584).

## Data processing and analyses

Count data were divided by the number of pullets (unit: count/ 5-minutes/ pullet). Data points from 20 WoA were excluded due to technical issues resulting in the loss of videos from one complete treatment group.

Statistical analyses were done in R, version 4.3.2. using generalized linear mixed models with the Tweedie distribution family (package: ‘glmmTMB’, link = “log”) to fit continuous, zero-inflated data. Fixed effects were feeding regimen (1 degree of freedom [df]), age at PS (1 df), age (9 df for aggression, otherwise 6 df), and all two-way interactions. Random effects accounted for the split-plot design (room by age at PS), the repeated measure within age (age nested in pen), and the effect of the room (pen nested in room). Abnormal pecking and locomotory behavior were analysed for data from weeks 15-22, aggressive behavior for weeks 15-25.

Data for abnormal pecking and aggressive behavior were additionally analyzed for R pullets exclusively to assess an effect of meals (pre vs post: 15-19 WoA, as 20 WoA was previously excluded). These analyzes included time relative to meals (1 df), age (4 df), and their interaction as fixed effects, and pen nested in room as the random effect. To reduce overdispersion, the dispersion parameter of the Tweedie distribution was modelled as a function of age.

Model fit was evaluated through the visual inspection of residual homoscedasticity and normality. Alpha level was set a 5%. Due to limited sample size, pairwise comparisons of significant interactions were assessed visually (packages: ‘emmeans’ and ‘ggplot2’) to avoid compromising statistical power and estimate reliability.

## Results and discussion

**Fig. 1:**
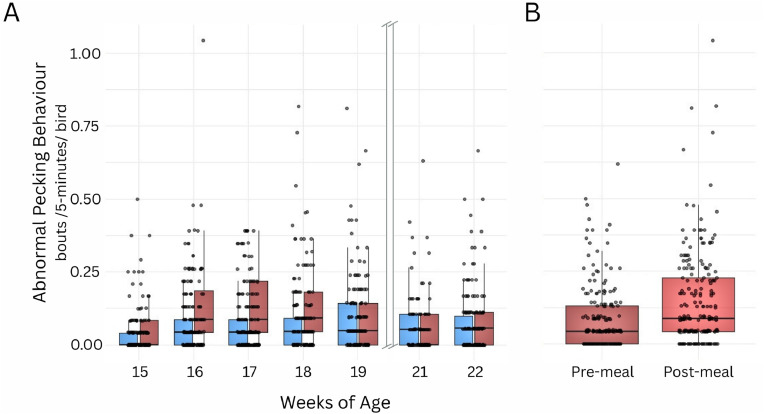
**Frequency of abnormal pecking behavior** (raw data). Blue represents the ad libitum fed birds and red represents restricted birds. **A**: Interactive between feeding regimen and age. **B**: Effect (statistical tendency) of time relative to feeding for restricted birds.

Abnormal pecking was affected by age (χ^2^ = 23.33, df = 6, *P* = 0.0006) and the feeding-by-age interaction of (χ^2^ = 25.50, df = 6, *P* = 0.0002; Figure 1A), but not by feeding (χ^2^ = 1.09, df = 1, *P* = 0.297) or age at PS (χ^2^ = 1.52, df = 1, *P* = 0.218). Frequencies were higher for R pullets at 16, 17, and 18 WoA. In R pullets, abnormal pecking tended to be more frequent post-meal than pre-meal (χ^2^ = 3.42, df = 1, *P* = 0.06; Figure 1B) and more frequent at 16 to 18 WoA compared to 15 or 19 WoA (χ^2^ = 17.70, df = 4, *P* = 0.001).

**Fig. 2:**
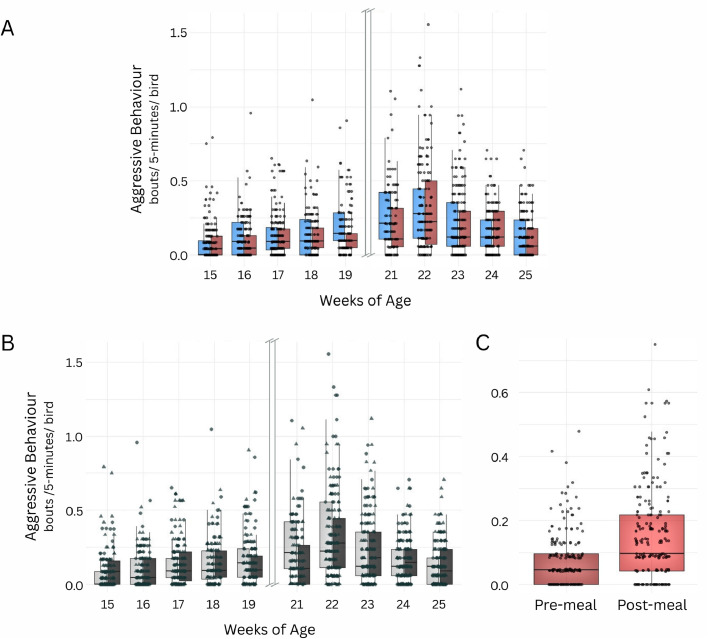
**Frequency of aggressive behavior** (raw data). Blue (left bars) indicates ad libitum fed birds, red (right bars) restricted birds, gray (left bars) represents photostimulation at 18 weeks of age (WoA), and black (right bars) represents photostimulation at 20 WoA. **A**: Interactive between feeding regimen and age. **B**: Interaction between age at photostimulation and age. **C**: Effect of time relative to feeding for restricted birds.

Aggressive behavior was not explained by feeding (χ^2^ = 0.21, df = 1, *P* = 0.650), but by age (χ^2^ = 135.06, df = 9, *P* < 0.0001), age at PS (χ^2^ = 4.55, df = 1, *P* = 0.033), and the feeding-by-age (χ^2^ = 20.28, df = 9, *P* = 0.016; Figure 2A) and age at PS-by-age interaction (χ^2^ = 23.86, df = 9, *P* = 0.005; Figure 2B). Aggression increased with age in all groups but increased faster in AL and early-PS pullets. Late-PS and R pullets caught up by 22 WoA, before aggression declined in all groups. Aggression in R pullets was more frequent post-meals than pre-meals (χ^2^ = 7.62, df = 1, *P* = 0.006; Figure 2C), but not affected by age (χ^2^ = 6.61, df = 4, *P* = 0.158).

Aerial locomotion did not appear affected by the main effects (feeding: χ^2^ = 0.003, df = 1, *P* = 0.954; age at PS: χ^2^ = 0.26, df = 1, *P* = 0.609; age: χ^2^ = 8.90, df = 6, *P* = 0.179). Despite the absence of a clear pattern, the age at PS-by-age interaction was statistically significant (χ^2^ = 15.51, df = 6, *P* = 0.017).

Ramp use appeared independent of the main effects (feeding: χ^2^ = 1.23, df = 1, *P* = 0.268; age at PS: χ^2^ = 0.10, df = 1, *P* = 0.752; age: χ^2^ = 9.93, df = 6, *P* = 0.127). Test statistics suggested an age at PS-by-age interaction (χ^2^ = 23.48, df = 6, *P* = 0.0007), though there was no apparent pattern discernible.

In this study, puberty was defined as the onset of lay, and group-level reproductive development was inferred from the age at 50% production (first to last [WoA]: AL_early-PS: 20.47, AL_late-PS: 20.98, R_early-PS: 21.02, R_late-PS: 22.21). For context, Lohmann LSL-Lite typical reach 50% production at 21.4 – 22.9 WoA (see Lohmann Tierzucht’s Management Guide Alternative Systems at https://lohmann-breeders.com/media/2021/03/LTZ_MG_management-systems_EN.pdf).

Feed deprivation can negatively impact animal welfare, resulting in abnormal behavior (Duncan, 2019). Here, feed restriction temporarily increased abnormal pecking behavior. Abnormal pecking and aggression were more frequent post- than pre-meals, suggesting frustration after an unsatisfactory meal. Abnormal pecking did return to lower frequencies before feed restriction was lifted, suggesting that: 1) pullets habituated to the low feed quantity and not longer perceived it as aversive, 2) feed restriction was only aversive during the specific ages, possibly associated with changes in growth rate, or 3) R pullets did not have enough energy to perform unwanted behaviours as feed restriction continued, masking the effect. Explanations one and two indicate that the impact on welfare was present but limited. It is unclear whether this temporary frustration would result in long-term effects. There are examples of enhanced stress resilience following early-life stress (in mice: Santarelli et al., 2017) as well as long-term detrimental effects from adverse conditions during adolescence, such as sustained changes in fear-related behavior and HPA-axis function (in chickens: Ericsson et al., 2016).

Aggressive behavior was not increased by restricted feeding, on the contrary, aggression was more frequent in AL pullets at certain ages. In all groups, aggression peaked at 22 WoA before decreasing. The treatment groups entering puberty first (AL and early-PS) displayed a steeper increase in aggression, indicating an association with sexual maturation. Dominance in layers has previously been associated with characteristics of their secondary sexual ornaments (comb and wattle) (O’Connor et al., 2011). As the development of these secondary sexual ornaments coincides with the reproductive development, the increase in averse social behavior during puberty might be a natural consequence.

Locomotory energy-expenditure was not affected by restricted feeding, suggesting that this level of restriction provided sufficient energy for normal vertical locomotion. These findings provide a counter to the notion that negative welfare impacts, such as abnormal behavior or aggression, were simply masked by reduced energy availability.

Lohmann LSL lite pullets subjected to a quantitative feed restriction of 20% displayed indicators of frustration with a temporary increase in abnormal pecking, mainly after meals. The effect disappeared before feed restriction was lifted, though long-term effects are unknown. Aggression increased throughout puberty and decreased after reaching maturity, suggesting a link between sexual maturation and the establishment of the pecking order. We found no evidence for a shift of vertical locomotion from high- to low-energy modes. Based on the data presented here, we propose that quantitative feed restriction of layer pullets in research should be implemented with meticulous monitoring of body weight and plumage, and encourage the investigation of potential long-term effects on latent feather-pecking risk and late-life bone health.

## Declaration of generative AI and AI-assisted technologies in the manuscript preparation process

During the preparation of this work the authors used Microsoft Copilot and Grammarly in order to troubleshoot R code syntax and grammar. After using this tool/service, the authors reviewed and edited the content as needed and take full responsibility for the content of the published article.

### Data availability statement

The data, ethogram, and R script supporting the findings presented here are publicly available in the Borealis data repository, the Canadian Dataverse Repository, at https://doi.org/10.5683/SP3/EUQVJU.

## CRediT authorship contribution statement

**Ana K. Rentsch:** Conceptualization, Methodology, Formal analysis, Investigation, Data curation, Writing – original draft, Writing – review & editing, Visualization. **Sachin Gautamadasa:** Methodology, Validation, Investigation, Data curation. **Charlene Hanlon:** Conceptualization, Writing – review & editing, Project administration, Funding acquisition. **Grégoy Y. Bédécarrats:** Conceptualization, Resources, Writing – review & editing, Supervision, Project administration, Funding acquisition.

## Disclosures

The authors declare the following financial interests/personal relationships which may be considered as potential competing interests:

Dr. Charlene Hanlon reports financial support was provided by Egg Industry Center. Dr. Gregoy Bedecarrats reports financial support was provided by Egg Farmers of Canada. Dr. Gregoy Bedecarrats reports financial support was provided by Ontario Ministry of Agriculture Food and Rural Affairs. Dr. Ana Rentsch reports financial support was provided by Foundation for Food and Agriculture Research. If there are other authors, they declare that they have no known competing financial interests or personal relationships that could have appeared to influence the work reported in this paper.
